# Examining glycation as a mediator linking bullying to psychotic experience and depressive symptom in adolescents

**DOI:** 10.1038/s41380-026-03521-7

**Published:** 2026-02-27

**Authors:** Mitsuhiro Miyashita, Zui C. Narita, Jordan Devylder, Syudo Yamasaki, Shuntaro Ando, Kazuya Toriumi, Satoshi Yamaguchi, Miharu Nakanishi, Mariko Hosozawa, Shinsuke Koike, Kazuhiro Suzuki, Kaori Baba, Junko Niimura, Naomi Nakajima, Deidre M. Anglin, Gemma Knowles, Craig Morgan, Marcus Richards, Mariko Hiraiwa-Hasegawa, Toshi A. Furukawa, Kiyoto Kasai, Atsushi Nishida, Makoto Arai

**Affiliations:** 1 Unit for Mental Health Promotion, Research Center for Social Science & Medicine, Tokyo Metropolitan Institute of Medical Science, Tokyo, Japan; 2 Department of Behavioural Medicine, National Institute of Mental Health, National Centre of Neurology and Psychiatry, Tokyo, Japan; 3 Silver School of Social Work, New York University, New York, United States of America; 4 Department of Child Neuropsychiatry, Graduate School of Medicine, The University of Tokyo, Tokyo, Japan; 5 Schizophrenia Research Project, Department of Psychiatry and Behavioral Sciences, Tokyo Metropolitan Institute of Medical Science, Tokyo, Japan; 6 Department of Psychiatric Nursing, Tohoku University, Sendai-shi, Miyagi Japan; 7 Department of Public Health and Primary Care, Leiden University Medical Center, Leiden, Zuid-Holland the Netherlands; 8 Research Center for Social Science & Medicine, Tokyo Metropolitan Institute of Medical Science, Tokyo, Japan; 9 Institute for Global Health Policy Research (iGHP), Bureau of International Health Cooperation, National Center for Global Health and Medicine, Tokyo, Japan; 10 The International Research Center for Neurointelligence (WPI-IRCN), The University of Tokyo Institutes for Advanced Study (UTIAS), Tokyo, Japan; 11 University of Tokyo Institute for Diversity & Adaptation of Human Mind (UTIDAHM), Tokyo, Japan; 12 Center for Evolutionary Cognitive Sciences, Graduate School of Art and Sciences, The University of Tokyo, Tokyo, Japan; 13 Department of Community Mental Health, Shinshu University School of Medicine, Matsumoto, Japan; 14 Department of Psychiatry, Shinshu University School of Medicine, Matsumoto, Japan; 15 Women’s Health and Midwifery, Graduate School of Nursing Science, St.Luke’s International University, Tokyo, Japan; 16 Department of Psychology, The City College of New York, City University of New York, New York, NY United States of America; 17 The Graduate Center, City University of New York, New York, NY United States of America; 18 ESRC Centre for Society and Mental Health, Institute of Psychiatry, Psychology, and Neuroscience, King’s College London, London, United Kingdom; 19 Health Service and Population Research Department, Institute of Psychiatry, Psychology, and Neuroscience, King’s College London, London, United Kingdom; 20 Emeritus Professor of Psychology, Unit for Lifelong Health and Ageing at UCL, University College London, London, United Kingdom; 21 School of Advanced Science, SOKENDAI (Graduate University for Advanced Studies), Hayama, Kanagawa Japan; 22 Kyoto University Office of Institutional Advancement and Communications, Kyoto, Japan

**Keywords:** Schizophrenia, Molecular biology

## Abstract

Bullying is a critical social stressor with long-lasting adverse impacts on mental health throughout life. Identifying biological mediators between bullying and subsequent mental health problems could help mitigate the long-term negative impact of bullying. However, such biological mediators have yet to be identified. This study assessed the mediating role of pentosidine, a representative glycation biomarker (i.e., pro-inflammatory aging compounds), between bullying and mental health problems among adolescents. This prospective, population-based cohort study (Tokyo Teen Cohort) included 3,158 participants. Causal mediation analysis was performed to test whether pentosidine at age 14 mediates the association between bullying at age 12 and subsequent mental health problems including psychotic experiences and depressive symptoms at age 16. Among the 3,158 adolescents aged 12 years (female, 46.9%), 473 (15.0%) were classified as being bullied. Bullying was associated with higher pentosidine levels (adjusted *β* [95% confidence interval], 0.27 [0.14–0.41]: *P* < 0.001) at age 14, and pentosidine at age 14 was associated with psychotic experiences (adjusted *β* [95% confidence interval], 0.02 [0.001–0.03]: *P* < 0.01) and depressive symptoms (adjusted *β* [95% confidence interval], 0.21 [0.09–0.32]; *P* < 0.001) at age 16. Pentosidine mediated 28.0% and 19.2% of the association between bullying and psychotic experiences and depressive symptoms, respectively. The mediating role of pentosidine was consistent across sexes. Higher levels of pentosidine possibly caused by bullying suggest that accelerated aging may begin from adolescence partly due to social stress. Future research should explore whether reducing pentosidine mitigates the adverse impact of bullying on subsequent mental health problems.

## Introduction

Social stress and adverse experiences in childhood and adolescence put youth at profoundly high risk for subsequent psychosis [1] and depression [2], with long-term impacts persisting into adulthood. These adverse experiences encompass a range of factors such as neglect and exposure to violence, and may account for 38% and 21% of psychotic [1] and depressive [2] disorders, respectively.

Bullying victimization is a widespread and salient social stressor during adolescence, with a global prevalence of approximately 11% [3]. Bullying victimization has been robustly linked to later mental health problems, including emotional problems and paranoid thoughts [4]. Moreover, bullying victimization profoundly impacts the increased risks of psychosis- [5] and depression- [6] related outcomes. Given this evidence, addressing the long-term adverse impact of bullying on subsequent mental health problems remains a focus of interventions, although bullying is difficult to effectively target. A complementary strategy may be to prevent subsequent mental health problems by targeting mediating biological mechanisms. However, these biological mediators have not yet been identified.

Advanced glycation end-products (AGEs) are pro-inflammatory and pro-oxidant compounds formed by a nonenzymatic glycation reaction between sugars, proteins, and lipids. Pentosidine is a representative biomarker of AGEs that accumulates with aging [7], contributing to cardiovascular disease and all-cause mortality in diabetes [8]. This highlights the critical role of pentosidine in accelerating aging. Additionally, pentosidine is an upstream compound that promotes the release of inflammatory cytokines, including interleukin-6, a well-established biomarker implicated in psychotic [9] and depressive [10] disorders. Pentosidine is also involved in oxidative stress [11], another important molecular mechanism related to psychotic [12] and depressive disorders [13]. Furthermore, pentosidine has been linked to white matter disruptions [14] and cognitive impairment [15], which are also implicated in psychotic [16] and depressive disorders [17], suggesting that pentosidine potentially contributes to structural and functional brain abnormalities. Collectively, these findings suggest that pentosidine may affect mental health by inducing structural and functional brain alterations through inflammation, oxidative stress, and accelerated aging. While higher levels of pentosidine are known to be caused by abnormal glucose metabolism, including diabetes, or by renal dysfunction with aging, no studies have investigated whether social stress—such as bullying—is associated with subsequent higher pentosidine levels. Moreover, growing evidence indicates that pentosidine is associated with schizophrenia [18], a finding consistently reported in adult patients [19], and that depressive symptoms and disorders have been linked to AGEs in adults [20]. However, the longitudinal relationship between pentosidine and subsequent psychosis- and depression-related outcomes remains unclear during adolescence.

In this study, we tested the hypothesis that bullying victimization (age 12) may lead to higher levels of pentosidine in adolescence (age 14) and that pentosidine may mediate the relationship between bullying and subsequent psychotic experiences and depressive symptoms (age 16). Our causal mediation analysis enabled us to elucidate the prospective association between bullying, pentosidine, and subsequent psychotic experiences and depressive symptoms within a causal inference framework [21].

## Materials and methods

### Study design and participants

This study used data from the Tokyo Teen Cohort (TTC), a prospective, population-based cohort study. Children born between September 1, 2002, and August 31, 2004, residing in three metropolitan municipalities (Setagaya, Mitaka, and Chofu) in Japan, were randomly recruited at the age of 10 years (October 2012 to January 2015) using the basic resident register [22]. Overall, 3,171 child-parent pairs participated at age 10, with 3,007, 2,667, and 2,616 pairs continuing at ages 12 (follow-up rate: 94.8%), 14 (follow-up rate: 84.1%), and 16 (follow-up rate: 82.5%), respectively. The exclusion criteria for this study included (1) antipsychotic medication use at age 12 or 14 and (2) severe physical illnesses such as ulcerative colitis at age 12 or 14, both of which could substantially affect pentosidine measurement. Thirteen participants were excluded, resulting in a final sample of 3,158 participants, all of whom were analyzed using the imputation methods (described below). The Ethics Committees of the Tokyo Metropolitan Institute of Medical Science (#12-35), the University of Tokyo (#10057), and SOKENDAI (the Graduate University for Advanced Studies; #2012002) approved the TTC study protocol. The parents provided written informed consent before their children’s participation. All methods were performed in accordance with the Declaration of Helsinki and relevant guidelines and regulations.

### Measures

#### Bullying victimization (Exposure)

Bullying victimization at age 12 was assessed by parents using the Strengths and Difficulties Questionnaire (SDQ), which has previously been translated and validated in Japanese [23]. The SDQ includes a specific item that evaluates peer relationship problems, which has been validated as a reliable measure for detecting bullying victimization [24]. Parents were asked to respond to a question regarding whether their child had been the target of bullying or teasing by other children, with the following three options: “not applicable,” “somewhat applicable,” or “applicable.” Responses of “somewhat applicable” or “applicable” were categorized as indicating that the child was a victim of bullying [25].

#### Urine pentosidine levels (Mediator)

Urine samples were collected early in the morning from participants at ages 12 and 14. Samples were promptly frozen after collection and maintained in frozen storage until analysis to inhibit the metabolism and de novo synthesis of pentosidine in urine. These urine samples were submitted to LSI Medience Corporation, a clinical laboratory testing company in Tokyo, Japan, and urine pentosidine levels were determined using high-performance liquid chromatography.

#### Psychotic experiences (Outcome)

We employed the Adolescent Psychotic-like Symptom Screener (APSS), a validated seven-item self-report questionnaire, to assess psychotic experiences at age 16 [26]. The Japanese version of the APSS is a widely used measure of psychotic experiences in Japan. Participants responded to each item using the following three options: “yes, definitely,” “maybe,” and “no, never,” scored as 1, 0.5, and 0 points, respectively. Scores were summed to generate a total score (range, 0–7), with higher scores indicating more severe psychotic experiences. In this study, the internal consistency of the APSS was acceptable, with a Cronbach’s alpha of 0.71. Psychotic experiences at age 12 were evaluated using items from the schizophrenia section of the Diagnostic Interview Schedule for Children [27].

#### Depressive symptoms (Outcome)

Depressive symptoms were assessed using the Short Mood and Feelings Questionnaire (SMFQ) at ages 12 and 16. The SMFQ includes 13 self-report questions rated on a 3-point Likert scale. Scores ranged from 0 to 26, with higher scores indicating more severe depressive symptoms. The SMFQ is widely recognized for its reliability and validity in assessing depressive symptoms in both clinical and community settings [28, 29]. In this study, the internal consistency of the SMFQ was strong, with a Cronbach’s alpha of 0.92.

#### Covariates

We selected covariates at age 12 based on the modified disjunctive cause criterion to adjust for potential causes of the exposure, outcome, or both, excluding instrumental variables and including covariates that acted as proxies for unmeasured variables that are common causes of both the exposure and the outcome [30]. The model included variables at age 12 that meet this criterion, namely age (months, continuous), sex (dichotomous), body mass index (BMI, continuous) [31], Intelligence Quotient (IQ, continuous) [32], and household income (JPY, < 5,000,000, 5,000,000–9,999,999, or ≥ 10,000,000, categorical) [33], loneliness (dichotomous, never vs. sometimes or always) [34, 35], physical punishment (dichotomous, never or rarely vs. sometimes, often, or always) [36], relationships with mother, father, and friends (all continuous) [37], neighborhood cohesion (continuous) [38, 39], gender nonconforming behavior (dichotomous, not at all vs. somewhat, sometimes, or often) [37], and problematic internet use (continuous) [40]. Per analysis, we included the baseline level of pentosidine and each mental health issue at age 12, thereby mitigating the possibility of reverse causation. Regarding the details on the measurement of covariates, loneliness was evaluated using a relevant item from the SMFQ [41]. The relationships with mother, father, and friends were evaluated using the Network of Relationships Inventory, where a higher score indicates better relationships [42]. Neighborhood cohesion was assessed using the Neighborhood Collective Efficacy scale [43], where a higher total score indicates greater neighborhood cohesion. Gender nonconforming behavior was assessed using the Youth Self Report [44], which asked if adolescents behaved like the opposite gender. Problematic internet use was evaluated via the modified Compulsive Internet Use Scale, where a greater score suggests a more problematic use of the internet [45].

#### Statistical analysis

All analyses were conducted using R 4.4.1. The R code used in this study is available from the corresponding author upon reasonable request. Missing data were handled using random forest imputation [46]. This non-parametric approach was selected because it can accommodate mixed-type data while capturing complex non-linear relationships between variables. The imputation procedure included all relevant variables. We first examined whether bullying victimization at age 12 was associated with pentosidine at age 14. We accounted for the aforementioned set of covariates, including the baseline level of pentosidine at age 12. We fitted the following three regression models: Model 1 adjusted for age, sex, BMI, and pentosidine at age 12; Model 2 adjusted for the variables included in Model 1, IQ, and household income; and Model 3 adjusted for the variables included in Model 2, loneliness, physical punishment, relationships with mother, father, and friends, neighborhood cohesion, gender nonconforming behavior, and problematic internet use.

Next, we examined whether pentosidine at age 14 was associated with psychotic and depressive symptoms at age 16. We accounted for the aforementioned set of covariates, including the baseline level of psychotic or depressive symptoms at age 12. Again, we fitted the following three regression models: Model 1 adjusted for age, sex, BMI, and psychotic or depressive symptoms at age 12 per analysis; Model 2 adjusted for the variables included in Model 1, IQ, and household income; and Model 3 adjusted for the variables included in Model 2, loneliness, physical punishment, relationships with mother, father, and friends, neighborhood cohesion, gender nonconforming behavior, and problematic internet use.

Finally, we evaluated the mediating role of pentosidine at age 14 in the association between bullying victimization at age 12 and psychotic and depressive symptoms at age 16. We used the CMAverse package [47] and accounted for exposure-mediator interaction that likely exists in real-world scenarios, thereby capturing the dynamics of mediation [21]. We accounted for the aforementioned set of covariates, including the baseline level of pentosidine and psychotic or depressive symptoms at age 12 per analysis, i.e., we adjusted for both the baseline level of mediator and outcome to mitigate the possibility of reverse causation. This approach enabled us to obtain total, pure direct, and total indirect effects as well as proportion mediated. Confidence intervals (CIs) for total, pure direct, and total indirect effects were calculated using the delta method [48]. We conducted three sensitivity analyses. First, we excluded loneliness from the model to see how the results would change, given that this variable was assessed using one of the items from the SMFQ, which was also used to evaluate depressive symptoms. Second, we evaluated pentosidine as a dichotomous variable by categorizing participants into high and low pentosidine groups based on the median value. Third, we further examined whether the associations were consistent when psychotic experiences and depressive symptoms at age 16 were dichotomized. Although dichotomizing continuous variables may lead to information loss and potentially misleading inferences [49–54], we conducted this analysis solely as a sensitivity analysis. For the presence of psychotic experiences, we defined psychotic experiences as selecting “yes, definitely” for at least one item on the APSS [26]. Depressive symptoms were dichotomized with a cut-off score of eight on the SMFQ [55]. Furthermore, we conducted sex-stratified mediation analysis to determine the degree of consistency of the mediating role of pentosidine.

## Results

Overall, 3158 participants (mean age, 146 months; female, 46.9%) were included in this study at age 12. Baseline characteristics of the participants with and without bullying victimization are summarized in Table 1. Participants who experienced bullying victimization were more likely to be male, have a low household income, feel lonely, experience physical punishment, exhibit gender nonconforming behavior, engage in a high degree of problematic internet use, and exhibit severe depressive symptoms. Higher levels of pentosidine (median [interquartile range (IQR)], 5.90 [4.90–7.10] pmol/mg*Cr) were observed in participants with bullying victimization than in those without bullying victimization (median [IQR], 5.60 [4.70–6.70] pmol/mg*Cr). Other characteristics showed no substantial differences across the groups. Psychotic experiences showed a positively skewed distribution, with most participants reporting no symptoms.

**Table 1:** Baseline characteristics of the study population at age 12.

		Bullying victimization		
	Overall (N = 3158)	No (N = 2514)	Yes or somewhat yes (N = 473)	Missing (N = 171)
Age, mean (SD), m	146 (3.30)	146 (3.28)	146 (3.40)	146 (3.27)
Missing, n (%)	4 (0.1)	2 (0.1)	1 (0.2)	1 (0.6)
Gender, n (%)				
Female	1480 (46.9)	1240 (49.3)	167 (35.3)	73 (42.7)
Male	1678 (53.1)	1274 (50.7)	306 (64.7)	98 (57.3)
BMI, median (IQR)	17.5 (16.2, 19.1)	17.4 (16.2, 19.0)	17.9 (16.3, 19.6)	17.4 (15.5, 19.0)
Missing, n (%)	614 (19.4)	378 (15.0)	70 (14.8)	166 (97.1)
IQ, mean (SD)	110 (14.9)	111 (14.6)	107 (15.8)	103 (11.0)
Missing, n (%)	628 (19.9)	390 (15.5)	72 (15.2)	166 (97.1)
Household income, JPY, n (%)				
< 5,000,000	446 (14.1)	357 (14.2)	88 (18.6)	1 (0.6)
5,000,000 to 9,999,999	1295 (41.0)	1076 (42.8)	217 (45.9)	2 (1.2)
≥ 10,000,000	680 (21.5)	589 (23.4)	90 (19.0)	1 (0.6)
Missing	737 (23.3)	492 (19.6)	78 (16.5)	167 (97.7)
Loneliness, n (%)				
Never	2119 (67.1)	1826 (72.6)	290 (61.3)	3 (1.8)
Sometimes or always	392 (12.4)	287 (11.4)	103 (21.8)	2 (1.2)
Missing	647 (20.5)	401 (16.0)	80 (16.9)	166 (97.1)
Physical punishment, n (%)				
Never or rarely	1832 (58.0)	1579 (62.8)	251 (53.1)	2 (1.2)
Sometimes, often, or always	904 (28.6)	718 (28.6)	184 (38.9)	2 (1.2)
Missing	422 (13.4)	217 (8.6)	38 (8.0)	167 (97.7)
Relationship with mother, median (IQR)	4.00 (3.11, 4.67)	4.00 (3.11, 4.67)	3.89 (3.00, 4.67)	3.89 (3.11, 4.67)
Missing, n (%)	404 (12.8)	336 (13.4)	57 (12.1)	11 (6.4)
Relationship with father, median (IQR)	3.67 (2.67, 4.44)	3.67 (2.67, 4.44)	3.44 (2.56, 4.44)	3.78 (2.78, 4.47)
Missing, n (%)	404 (12.8)	336 (13.4)	57 (12.1)	11 (6.4)
Relationships with friends, median (IQR)	3.56 (2.89, 4.22)	3.56 (2.89, 4.22)	3.56 (2.78, 4.22)	3.44 (2.89, 4.22)
Missing, n (%)	404 (12.8)	336 (13.4)	57 (12.1)	11 (6.4)
Neighborhood cohesion, mean (SD)	12.9 (2.89)	12.9 (2.91)	12.8 (2.83)	13.1 (2.73)
Missing, n (%)	176 (5.6)	141 (5.6)	31 (6.6)	4 (2.3)
Gender nonconforming behavior, n (%)				
Not at all	1808 (57.3)	1448 (57.6)	254 (53.7)	106 (62.0)
Somewhat, sometimes, or often	703 (22.3)	539 (21.4)	124 (26.2)	40 (23.4)
Missing	647 (20.5)	527 (21.0)	95 (20.1)	25 (14.6)
Problematic internet use, median (IQR)	3.00 (0.00, 6.00)	2.00 (0.00, 6.00)	4.00 (1.00, 7.00)	5.00 (5.00, 6.00)
Missing, n (%)	202 (6.4)	28 (1.1)	8 (1.7)	166 (97.1)
Pentosidine, pmol/mg・Cr, median (IQR)	5.70 (4.70, 6.80)	5.60 (4.70, 6.70)	5.90 (4.90, 7.10)	6.20 (4.90, 7.20)
Missing, n (%)	1573 (49.8)	1194 (47.5)	212 (44.8)	167 (97.7)
Psychotic experiences, median (IQR)	0.00 (0.00, 1.00)	0.00 (0.00, 1.00)	0.00 (0.00, 1.00)	0.00 (0.00, 1.00)
Missing, n (%)	629 (19.9)	514 (20.4)	91 (19.2)	24 (14.0)
Depressive symptoms, median (IQR)	2.00 (0.00, 5.00)	2.00 (0.00, 5.00)	4.00 (1.00, 9.00)	4.00 (3.00, 9.00)
Missing, n (%)	688 (21.8)	433 (17.2)	89 (18.8)	166 (97.1)

We reported mean (SD) for normally distributed variables and median (IQR) for non-normally distributed variables.

*SD* standard deviation, *BMI* body mass index, *IQR* interquartile range, *IQ* intelligence quotient.

**Fig. 1:**
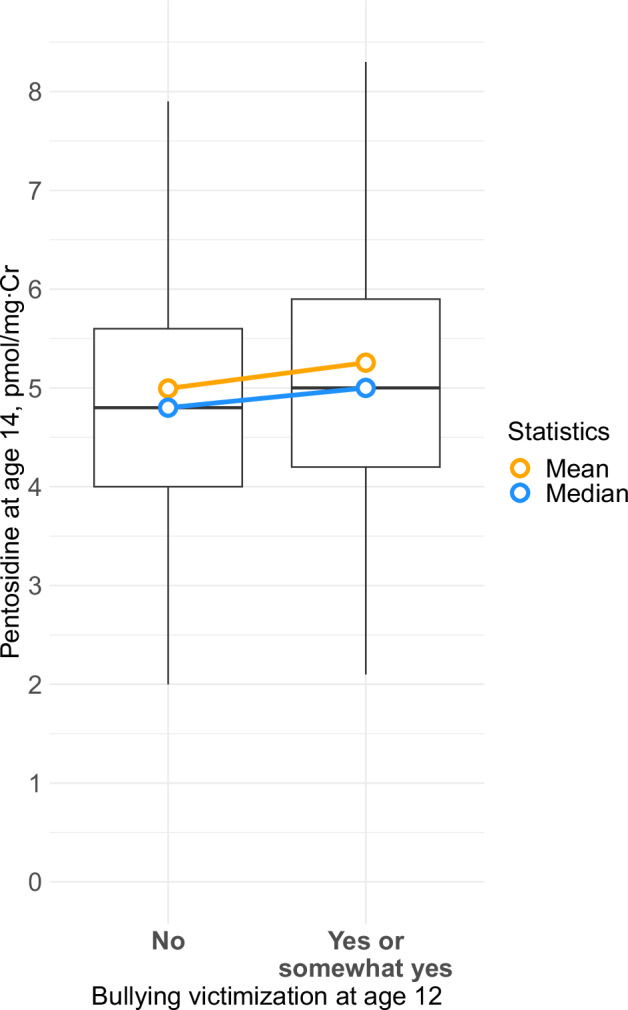
Urine pentosidine levels for participants with and without bullying victimization at age 14. Box-and-whisker plot of urine pentosidine levels for individuals with and without bullying victimization at age 12. The horizontal axis represents the two groups: with bullying (right) and without bullying (left), and the vertical axis shows the urine pentosidine levels. Each box indicates the interquartile range (IQR), with whiskers representing the minimum and maximum value within 1.5 times the IQR. The blue circles represent the median values for each group, while the orange circles indicate the mean values.

Figure 1 illustrates pentosidine levels at age 14. Additionally, higher levels of pentosidine (median [IQR], 5.00 [4.20–6.00] pmol/mg*Cr) were observed in participants with bullying victimization than in those without bullying victimization (median [IQR], 4.80 [4.00–5.70] pmol/mg*Cr).

### Bullying victimization and pentosidine

We assessed whether bullying victimization at age 12 contributed to higher levels of pentosidine at age 14. After adjusting for a comprehensive set of covariates, including the baseline level of pentosidine at age 12, bullying victimization at age 12 was significantly associated with pentosidine at age 14 (adjusted *β* [95% CI], 0.27 [0.14–0.41]; *P* < 0.001; Model 3 at Table 2). The findings were consistent across Models 1–3.

**Table 2:** Associations between bullying victimization at age 12 and subsequent pentosidine at age 14.

	Model 1		Model 2		Model 3	
Exposure	*β* [95% CI]	*P*	*β* [95% CI]	*P*	*β* [95% CI]	*P*
Bullying victimization						
No	0.00 (Reference)		0.00 (Reference)		0.00 (Reference)	
Yes or somewhat yes	0.31 [0.18, 0.45]	< 0.001	0.31 [0.17, 0.45]	< 0.001	0.27 [0.14, 0.41]	< 0.001

Missing data were handled using random forest imputation.

Model 1 adjusted for age, sex, BMI, and pentosidine at age 12.

Model 2 adjusted for age, sex, BMI, IQ, household income, and pentosidine at age 12.

Model 3 adjusted for age, sex, BMI, IQ, household income, loneliness, physical punishment, relationships with mother, father, and friends, neighborhood cohesion, gender nonconforming behavior, problematic internet use, and pentosidine at age 12.

*CI* confidence interval, *BMI* body mass index, *IQ* intelligence quotient.

### Pentosidine and mental health issues

We next examined whether pentosidine at age 14 was associated with psychotic experiences and depressive symptoms at age 16. After adjusting for a comprehensive set of covariates, including the baseline level of each mental health issue at age 12, pentosidine at age 14 was significantly associated with psychotic experiences (adjusted *β* [95% CI], 0.02 [0.001–0.03]; *P* < 0.01) and depressive symptoms (adjusted *β* [95% CI], 0.21 [0.09–0.32]; *P* < 0.001) at age 16 (Model 3, Table 3). For both psychotic experiences and depressive symptoms, the findings were similar across Models 1–3.

**Table 3:** Associations between pentosidine at age 14 and subsequent mental health issues at age 16.

	Model 1		Model 2		Model 3	
Outcomes	*β* [95% CI]	*P*	*β* [95% CI]	*P*	*β* [95% CI]	*P*
Psychotic experiences	0.02 [0.003, 0.03]	0.02	0.02 [0.002, 0.03]	0.03	0.02 [0.001, 0.03]	< 0.01
Depressive symptoms	0.22 [0.10, 0.34]	< 0.001	0.22 [0.10, 0.34]	< 0.001	0.21 [0.09, 0.32]	< 0.001

Missing data were handled using random forest imputation.

Model 1 adjusted for age, sex, BMI, and each mental health issue at age 12.

Model 2 adjusted for age, sex, BMI, IQ, household income, and each mental health issue at age 12.

Model 3 adjusted for age, sex, BMI, IQ, household income, loneliness, physical punishment, relationships with mother, father, and friends, neighborhood cohesion, gender nonconforming behavior, problematic internet use, and each mental health issue at age 12.

*CI* confidence interval, *BMI* body mass index, *IQ* intelligence quotient.

### Mediation by pentosidine of the association between bullying victimization and mental health issues

Finally, we tested whether pentosidine at age 14 mediated the association between bullying victimization at age 12 and psychotic experiences and depressive symptoms at age 16, adjusting for a comprehensive set of covariates, including the baseline level of pentosidine and each mental health issue at age 12. We estimated that pentosidine mediated 28.0% and 19.2% of the pathway from bullying victimization to psychotic experiences and depressive symptoms, respectively (Table 4). Significant total indirect effects were found for psychotic experiences (adjusted *β* [95% CI], 0.02 [0.005–0.03]) and depressive symptoms (adjusted *β* [95% CI], 0.15 [0.04–0.26]; Table 4). The sensitivity analysis excluding loneliness from the model yielded analogous results (Supplementary Table 1 in the online supplement). Evaluating pentosidine as a dichotomous variable based on the median value also produced similar results (Supplementary Table 2 in the online supplement). While examining depression as a dichotomous variable resulted in similar results, we did not find evidence for dichotomized psychotic epxeriences (Supplementary Table 3 in the online supplement). Sex-stratified analysis showed that the mediating role of pentosidine was consistent across both sexes (Supplementary Tables 4 and 5 in the online supplement).

**Table 4:** Mediating role of pentosidine at age 14 for the association between bullying victimization at age 12 and mental health issues at age 16.

Outcome	Exposure	TE *β* [95% CI]	PDE *β* [95% CI]	TIE *β* [95% CI]	*P* for TIE	PM %
Psychotic experiences	Bullying victimization					
	No	0.00 (Reference)	0.00 (Reference)	0.00 (Reference)		
	Yes or somewhat yes	0.07 [0.00, 0.13]	0.05 [−0.01, 0.11]	0.02 [0.005, 0.03]	< 0.01	28.0
Depressive symptoms	Bullying victimization					
	No	0.00 (Reference)	0.00 (Reference)	0.00 (Reference)		
	Yes or somewhat yes	0.72 [0.24, 1.21]	0.61 [0.12, 1.11]	0.15 [0.04, 0.26]	< 0.01	19.2

Missing data were handled using random forest imputation.

PM was calculated by TIE/TE.

The model adjusted for age, sex, BMI, IQ, household income, loneliness, physical punishment, relationships with mother, father, and friends, neighborhood cohesion, gender nonconforming behavior, problematic internet use, pentosidine, and each mental health issue at age 12.

CIs were computed using the delta method.

*TE* total effect, *PDE* pure direct effect, *TIE* total indirect effect, *PM* proportion mediated, *CI* confidence interval, *BMI* body mass index;, *IQ* intelligence quotient.

## Discussion

This study investigated the prospective associations between bullying victimization at age 12, pentosidine at age 14, and psychotic experiences and depressive symptoms at age 16. The results revealed that bullying victimization at age 12 was associated with pentosidine at age 14 and that pentosidine at age 14 mediated the association between bullying victimization at age 12 and subsequent psychotic experiences and depressive symptoms at age 16. These results remained robust and statistically significant after adjusting for multiple potential confounders, including those accounting for reverse causation.

Elevated levels of pentosidine, possibly caused by bullying victimization, could provide a potential mechanism through which accelerated aging may begin from adolescence, due in part to social stress. This analysis provides a conceptual demonstration that pentosidine may provide a biological mechanism linking bullying and similar exposures to mental health outcomes. Such biological mechanisms are clinically crucial but have been largely elusive in our understanding of the social etiology of psychiatric symptoms. This study provides the first evidence that pentosidine may serve as a potential biological mediator, which (if targeted clinically) could mitigate the negative impacts of bullying victimization on subsequent psychotic experiences and depressive symptoms. Randomized controlled trials, or if these are not feasible, observational studies emulating target trials, are warranted [56] to investigate whether reducing pentosidine levels could mitigate the adverse effects of bullying on subsequent mental health outcomes.

Lifestyle interventions, including healthy diet and exercise are inversely associated with psychosis [57] and depression [58, 59], although the biological mechanism remains unclear. Diet has been identified as a potential strategy to lower pentosidine levels [60]. Our previous study also supported the effect of exercise on reducing pentosidine [61]. Additionally, pentosidine may contribute to psychotic experiences and depressive symptoms by potentially influencing the brain structure and function through inflammation, oxidative stress, and accelerated aging. Ultimately, diet and exercise might improve psychotic experiences and depressive symptoms by counteracting the detrimental effects of pentosidine, namely suppressing inflammation, oxidative stress, and aging, providing a potentially low-risk and low-cost intervention approach. Examining the efficacy of targeted lifestyle interventions to reduce pentosidine may offer valuable insights for improving youth mental health. Direct pharmacological or biological approaches to modify pentosidine levels may also warrant further exploration.

Patients with schizophrenia have a life expectancy of more than 20 years shorter than that of the general population, and this trend has not improved recently [62]. Particularly, the mortality rate from cardiovascular disorders is the highest among all causes of death related to schizophrenia [63]. Similarly, depressive disorders have been reported to have many physical complications, including cardiovascular diseases. Additionally, people with depressive disorders have a higher mortality rate than those without depressive disorders [64]. Such evidence clearly indicates that aging is accelerated in both psychotic and depressive disorders. Pentosidine has also been known to be associated with aging [7] by acting as an upstream compound that promotes inflammatory response. Furthermore, pentosidine has been linked to increased mortality risk in individuals with diabetes [8] and chronic kidney disease [11], suggesting that pentosidine accelerates aging, potentially playing a critical role in the pathophysiology and disease progression. Notably, adverse childhood experiences, including bullying victimization, have been associated not only with long-term psychiatric outcomes such as psychosis and depression, but also with somatic outcomes including cardiovascular disease, diabetes, and cancer [65]. Given that pentosidine has been linked to biological aging, pentosidine may be involved as one potential shared biological pathway linking adverse experiences in childhood to both psychiatric and somatic outcomes. Therefore, pentosidine-targeted interventions may contribute not only to the prevention of subsequent mental health problems but also to addressing the subsequent physical health inequality.

For years, higher levels of pentosidine were thought to be the results of antipsychotic medication due to the cross-sectional association of antipsychotics with pentosidine in patients with chronic adult schizophrenia [66, 67], although the factors contributing to the higher levels of pentosidine during adolescence remained unknown. In this study, after rigorously excluding the influence of antipsychotic medication, bullying victimization emerged as a novel contributor to higher levels of pentosidine in adolescents. Future research is required to investigate whether other forms of social stress, racism [68], neighborhood ethnoracial diversity [69], and gender inequality [70], which have been connected to peer victimization and identified in other marginalized groups, similarly contribute to higher levels of pentosidine.

The specific molecular mechanism of increased levels of pentosidine due to bullying victimization remains to be clarified. One possible molecular explanation is a persistent and mild stress-related abnormality in glucose metabolism via hypothalamic-pituitary-adrenal axis disturbance. Another explanation might be mitochondrial dysfunction and oxidative stress caused by AGE precursor [71]. However, future basic and clinical studies are warranted to uncover the precise molecular basis of higher levels of pentosidine associated with social stress.

This study has several strengths. We used longitudinal data collected at three distinct time points and carefully selected potential confounders, enabling a robust examination of the causal relationships between bullying victimization, pentosidine, and subsequent psychotic and depressive symptoms. Additionally, the effect of antipsychotics on pentosidine was completely excluded in this study. However, this study also had some limitations. First, the assessment of bullying was based solely on parent reports via SDQ, and neither the severity nor the subjective impact on the participants was assessed. Incorporating multi-informant data, including bullying severity and adolescent self-reports, would allow for a more accurate estimation of pentosidine’s mediating role and may help identify subgroups for whom interventions targeting pentosidine could be particularly effective. Future research should aim to further clarify these issues. Second, factors mediating the relationship between bullying victimization and psychotic experiences and depressive symptoms may involve not only pentosidine but also other compounds generated by bullying. Third, while we assume biological mechanisms linking stress to mental health will be relatively consistent across settings, further investigations will be required to examine the generalizability of our findings beyond metropolitan Tokyo. Fourth, although bullying victimization showed total effects on continuous measures of psychotic experiences and depressive symptoms, this does not necessarily imply a clinically meaningful difference, given the small coefficient. Also, in the sensitivity analysis using dichotomized outcomes, we did not find evidence for an association with psychotic experiences. Analyzing continuous outcomes is statistically preferable because dichotomizing continuous variables may lead to information loss and misleading inferences, as multiple methodological papers have cautioned [49–54]. Therefore, the dichotomized analysis was used solely as a sensitivity analysis, and our findings regarding psychotic experiences should be interpreted primarily in terms of severity rather than presence. Finally, our causal mediation analysis relies on four assumptions: (i) no exposure-outcome confounding after the adjustments, ii) no mediator-outcome confounding after the adjustments, (iii) no exposure-mediator confounding after the adjustments, and (iv) no mediator-outcome confounders influenced by exposure [21, 48]. While we adjusted for a rich set of covariates, these assumptions were inherently unverifiable.

Higher levels of pentosidine possibly caused by bullying suggest that accelerated aging may begin from adolescence due in part to social stress. Furthermore, pentosidine may serve as a novel biological mediator of bullying with psychotic experiences and depressive symptoms. Future research should explore whether reducing pentosidine mitigates the long-term adverse impact of bullying on subsequent psychotic and depressive disorders.

## Supplementary information

Sensitivity analysis for mediation: excluding loneliness from the model.

Sensitivity analysis for mediation: evaluating pentosidine as a dichotomous variable.

Sensitivity analysis for mediation: evaluating psychotic experiences and depression as dichotomous variables.

Subgroup analysis: Female participants (N = 1480).

Subgroup analysis: Male participants (N = 1678).

## Data Availability

The datasets generated and analyzed during the current study are not publicly available but are available from the corresponding author upon reasonable request.
