# Supplementary material for: Examining glycation as a mediator linking bullying to psychotic experience and depressive symptom in adolescents
Source: Mol Psychiatry. 2026 Feb 27;31(7):3872–9. doi: 10.1038/s41380-026-03521-7 (PMC13268971; doi:10.1038/s41380-026-03521-7)
Supplement: Supplementary file 2 — Sensitivity analysis for mediation: evaluating pentosidine as a dichotomous variable. [file 41380_2026_3521_MOESM2_ESM.docx]

Supplementary Table 2. Sensitivity analysis for mediation: evaluating pentosidine as a dichotomous variable.

| Outcome | Exposure | TE  *β*  [95% CI] | PDE  *β*  [95% CI] | TIE  *β*  [95% CI] | *P* for TIE | PM  % |
| --- | --- | --- | --- | --- | --- | --- |
| Psychotic experiences | Bullying victimization |  |  |  |  |  |
|  | No | 0.00  (Reference) | 0.00  (Reference) | 0.00  (Reference) |  |  |
|  | Yes or somewhat yes | 0.07  [0.01, 0.14] | 0.06  [-0.01, 0.12] | 0.02  [0.00, 0.03] | 0.03 | 21.7 |
| Depressive symptoms | Bullying victimization |  |  |  |  |  |
|  | No | 0.00  (Reference) | 0.00  (Reference) | 0.00  (Reference) |  |  |
|  | Yes or somewhat yes | 0.77  [0.28, 1.26] | 0.64  [0.15, 1.13] | 0.13  [0.02, 0.24] | 0.02 | 16.6 |

TE, total effect; PDE, pure direct effect; TIE, total indirect effect; PM, proportion mediated; CI, confidence interval; BMI, body mass index; IQ, intelligence quotient.

Missing data were handled using random forest imputation.

PM was calculated by TIE/TE.

The model adjusted for age, sex, BMI, IQ, household income, physical punishment, relationships with mother, father, and friends, neighborhood cohesion, gender nonconforming behavior, problematic internet use, pentosidine, and each mental health issue at age 12.

CIs were computed using the delta method.
