# Supplementary material for: Examining glycation as a mediator linking bullying to psychotic experience and depressive symptom in adolescents
Source: Mol Psychiatry. 2026 Feb 27;31(7):3872–9. doi: 10.1038/s41380-026-03521-7 (PMC13268971; doi:10.1038/s41380-026-03521-7)
Supplement: Supplementary file 3 — Sensitivity analysis for mediation: evaluating psychotic experiences and depression as dichotomous variables. [file 41380_2026_3521_MOESM3_ESM.docx]

Supplementary Table 3. Sensitivity analysis for mediation: evaluating psychotic experiences and depression as dichotomous variables.

| Outcome | Exposure | TE  OR  [95% CI] | PDE  OR  [95% CI] | TIE  OR  [95% CI] | *P* for TIE | PM  % |
| --- | --- | --- | --- | --- | --- | --- |
| Psychotic experiences | Bullying victimization |  |  |  |  |  |
|  | No | 1.00  (Reference) | 1.00  (Reference) | 1.00  (Reference) |  |  |
|  | Yes or somewhat yes | 0.89  [0.61, 1.30] | 0.87  [0.59, 1.29] | 1.02  [0.96, 1.08] | 0.59 | NA^a^ |
| Depression | Bullying victimization |  |  |  |  |  |
|  | No | 0.00  (Reference) | 0.00  (Reference) | 0.00  (Reference) |  |  |
|  | Yes or somewhat yes | 1.43  [1.11, 1.84] | 1.35  [1.05, 1.73] | 1.06  [1.01, 1.12] | 0.02 | 19.3 |

TE, total effect; PDE, pure direct effect; TIE, total indirect effect; PM, proportion mediated; CI, confidence interval; BMI, body mass index; IQ, intelligence quotient.

Missing data were handled using random forest imputation.

The model adjusted for age, sex, BMI, IQ, household income, physical punishment, relationships with mother, father, and friends, neighborhood cohesion, gender nonconforming behavior, problematic internet use, pentosidine, and each mental health issue at age 12.

CIs were computed using the delta method.

^a^ Not estimable because the PDE and TIE were in opposite directions.
